# Maskless Lithography and *in situ* Visualization of Conductivity of Graphene using Helium Ion Microscopy

**DOI:** 10.1038/srep11952

**Published:** 2015-07-07

**Authors:** Vighter Iberi, Ivan Vlassiouk, X.-G. Zhang, Brad Matola, Allison Linn, David C. Joy, Adam J. Rondinone

**Affiliations:** 1 Center for Nanophase Materials Sciences, Oak Ridge National Laboratory, Oak Ridge, TN 37831, USA; 2 Energy and Transportation Science, Oak Ridge National Laboratory, Oak Ridge, TN 37831; 3 Department of Physics and Quantum Theory Project, University of Florida, Gainesville, FL 32611; 4 Department of Materials Science & Engineering, University of Tennessee Knoxville, TN 37996

## Abstract

The remarkable mechanical and electronic properties of graphene make it an ideal candidate for next generation nanoelectronics. With the recent development of commercial-level single-crystal graphene layers, the potential for manufacturing household graphene-based devices has improved, but significant challenges still remain with regards to patterning the graphene into devices. In the case of graphene supported on a substrate, traditional nanofabrication techniques such as e-beam lithography (EBL) are often used in fabricating graphene nanoribbons but the multi-step processes they require can result in contamination of the graphene with resists and solvents. In this letter, we report the utility of scanning helium ion lithography for fabricating functional graphene nanoconductors that are supported directly on a silicon dioxide layer, and we measure the minimum feature size achievable due to limitations imposed by thermal fluctuations and ion scattering during the milling process. Further we demonstrate that ion beams, due to their positive charging nature, may be used to observe and test the conductivity of graphene-based nanoelectronic devices *in situ*.

Scanning helium ion microscopy (SHIM) and ion milling (performed with the same instrument) has emerged as a tool capable of fabricating graphene devices^1^,^2^ with feature sizes down to ~5 nm^3^. Commercial SHIMs are now capable of ion milling at the nanoscale using either He^+^ or Ne^+^. Moreover, as a direct-fabrication method, He^+^ milling requires no resists, developer or wash to fabricate features yet can achieve feature sizes comparable to or smaller than EBL. The smaller interaction volume of the helium ion beam with the sample results in reduced proximity effects compared to EBL, and improved sputtering yield in the surface layer^1^. In our experiment, simple graphene structures consisting of a strip and a pad (Fig. 1) were fabricated using such direct-write lithography^1^ in a SHIM (see Methods section), with the graphene grounded to the instrument for charge compensation due to loss of secondary electrons. A square (or pad) is milled out leaving only a thin strip connecting the pad to the remaining graphene around the pad. The thin strip acts as a conductor between the surrounding graphene area and the milled pad.

By varying the length and width of the strip and then imaging the entire structure, we can directly observe how the electrical conductivity changes as a function of pad brightness. As the ion beam impinges on the pad, secondary electrons are ejected from the pad, yielding a significant net positive charge on the pad. Unlike in a scanning electron microscope (SEM) the positively charged ion beam is not capable of compensating for lost secondary electrons. While graphene’s work function can be tuned across the charge neutrality point by an electric field effect^4^ and doping^5^, this tunability can be achieved in SHIM by directly depleting electrons from the graphene through the loss of secondary electrons during imaging. Therefore if the pad in the center of the device is not grounded because the graphene strip is poorly conducting, then compensating electrons may not flow into the pad. This will cause a depression of the graphene work function and subsequent lower yield of secondary electrons, resulting in a darker image. This effect may be used to test conductivity of devices *in situ*, thereby allowing for the estimation of the quality of a graphene conductor. Furthermore, SHIM provides unprecedented high contrast images due to its shorter range and superior ion-generated secondary electron (iSE) yield^6^. Hence, electrical conductivity in the patterned graphene structures was explored *in situ* by varying the size and width of the connecting strip in each device.

## Results

**Figure 1:**
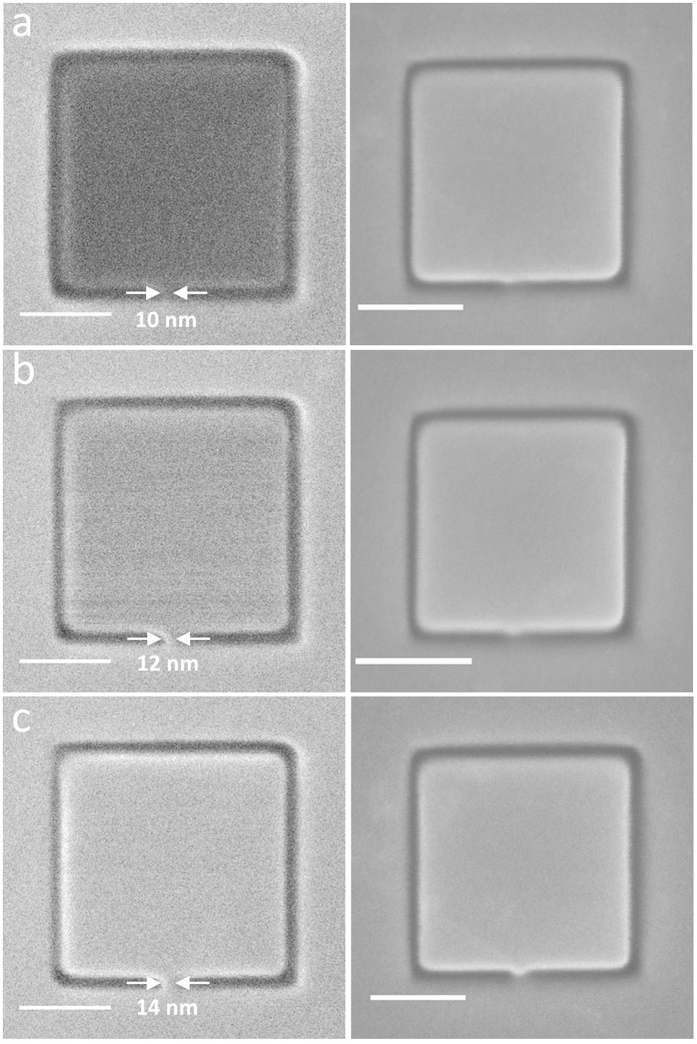
(Left panel) SHIM images of graphene-based pads that have been fabricated using direct-write He^+^ lithography. (Top left) Nonconducting graphene pad due to insufficient supply of electrons through the thin conducting graphene strip (~10 nm). (Middle left) Onset of slight conduction in graphene pad as the width of the conducting strip is increased to 12 nm. Thermal noise is also evident in the pad. (Bottom left) Fully conducting graphene pad with conducting strip width of 14 nm. (Right panel) SEM images of the exact same structures indicating insufficient electrons in the graphene pads (top and middle right) are compensated by the electron beam. Scale bar is 50 nm.

Figure 1 illustrates a series of iSE images of He^+^ milled graphene pads (left panel) that have been acquired following the fabrication process. Corresponding SEM images of the exact same devices (right panel) were used to directly compare the effects of imaging with a negatively charged beam. In Fig. 1a, the dark region in the iSE image indicates an area with low electron density. While the area surrounding the device remains bright, the low electron density region within the device suggests that the connecting strip (~10 nm) is not mediating the flow of sufficient electrons into the device. Furthermore, as the ion beam is raster-scanned over the device, the accumulation of positive charge on the surface precludes the ejection of secondary electrons energetic enough to be detected^7^. On the other hand, the SEM image does not indicate this effect due to the fact that the insufficient supply of electrons from the strip to the device is compensated by the negative charge of the electron beam. While a direct measurement of the current across the strip is not possible in this instrument, we can indirectly measure current across the strip. The compensation current to the stage and the beam current are measured directly; subtracting those estimates the secondary electron current. Most 30 keV He^+^ do not neutralize at the graphene due to their velocity, but neutralize deeper in the Si wafer. In general, these strips are compensating for lost secondary electrons, and conducting around 1 pA. The transition from bright to dark occurs when the strip is no longer capable of carrying 1 pA. As the width of the strip is increased to 12 nm (Fig. 1b), more electrons are conducted into the device and are sufficient enough to overcome the loss of secondary electrons due to the impinging ion beam. Consequently, the device is conducting and is demonstrated by the increase in brightness relative to the device in Fig. 1a.

Of particular interest is the subtle noise in the pad in Fig. 1b. This noise is ubiquitous under certain conditions and only occurs within the pad area of the image. This image noise may be attributed to thermal noise or vibrations in the graphene device which cause it to oscillate at its natural frequency. Theoretical simulations done previously by Sakhaee-Pour *et al.*^8^, and Ansari *et al.*^9^, have demonstrated that a 50 nm square SLG sheet has a natural frequency of about 3 GHz. Scaling this to a 500 nm square SLG would yield a natural frequency of about 30 MHz (or 33 ns period). Considering ways in which vibrations may impact electron transport and image brightness suggests a coupling of the vibrational modes of the device, the strip and the surrounding sheet. These coupled vibrations change the instantaneous elastic configuration of the strip, which appears as a disordered scattering potential for the electrons and leads to a change in the electrical conductivity of the strip^10^. This in turn causes variation in the electron flow across the strip which impacts the work function and brightness of the image. The longer period (μs) fluctuation can be explained in terms of beats between two degenerate free oscillation modes modified by the coupling to the strip, 
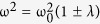
 where the coupling strength λ can be approximated by the size ratio between the strip and the pad, or λ = 10^−2^ which leads to a beat period of about 3 μs, within the detection range of the dwell time (50 μs). Also, this noise (at room temperature) may place a limit on the smallest feature sizes that can be used for graphene circuitry supported by SiO_2_, which in our case is ~10 nm. This noise does not represent the distribution of electrons within the pad – rather, this noise reflects the instantaneous conductivity of the strip at the moment that pixel is being collected. Figure 1c which corresponds to a strip width of 16 nm is fully bright. This indicates a sufficient supply of electrons from the surrounding graphene area into the milled pad, no noise, and hence, a fully conducting device.

**Figure 2:**
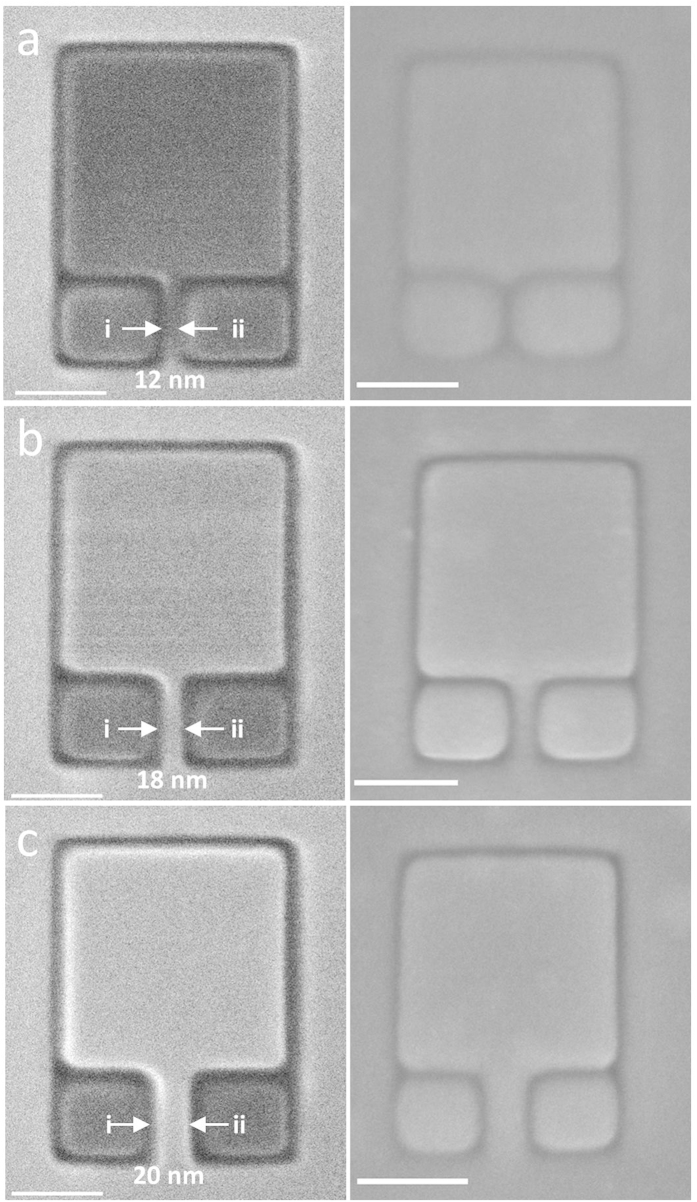
(Left panel) SHIM images of graphene-based pads with longer connecting strips that have been fabricated using direct-write He^+^ lithography. Areas (i) and (ii) are isolated graphene regions within the device. (Top left) Nonconducting graphene pad due to insufficient supply of electrons through the thin conducting graphene strip (~12 nm). (Middle left) Onset of slight conduction in graphene pad as the width of the conducting strip is increased to 18 nm. Thermal noise is also evident in the pad. (Bottom left) Fully conducting graphene pad with conducting strip width of 20 nm. (Right panel) SEM images of the exact same structures indicating that insufficient electrons in the graphene pads (top and middle) are compensated by the electron beam. Scale bar is 50 nm.

A second class of graphene devices was explored in order to investigate the impact of the length of the conducting strip. These devices were fabricated to include two smaller regions (i and ii) that have been completely electrically isolated in order create conducting-insulating composites. Similar to Fig. 1, the corresponding SEM images have been used for direct comparison (Fig. 2, right panel). Beam damage resulting from prolonged exposure of the graphene device to the electron beam is also evident. In Fig. 2a, the width of the connecting bridge is ~12 nm but due to the increased length and possibly the effect of vibrations, it appears to be nonconducting as was the device in Fig. 1a. Figure 2(b–c) shows the progression of the device towards conductivity (beginning at ~18 nm) while the sub regions i and ii constantly remain insulating. Similar to Fig. 1b, image noise just within the pad indicate thermal fluctuations in the device and suggest that the electrical properties of the strip strongly depend on its width. Avouris and co-workers^11^ studied the effect of decreasing the width of graphene nanoribbons fabricated with EBL on the electrical properties of field-effect transistor devices. The minimum graphene nanoribbon width achieved with their method was 20 nm. Their results showed that as the width of the nanoribbon decreased, its maximum resistivity increased at room temperature. This effect was attributed to scattering which occurred at the rough boundaries of the nanoribbon, and imperfections at the atomic scale. In our experiment, a minimum width of 10 nm for the shorter strip and 12 nm for the longer strip show the same effect on the conductivity of the graphene pads.

Concerning the distinct bright edges in Fig. 2b,c, these may be attributed to the topography and orientation of the milled edges in the device relative to the Everhart-Thornley (ET) secondary electron detector. Consequently, the emitted secondary electrons will not have an equal probability of being collected by each solid angle. Also, it should be noted that this *in situ* conductivity can be observed in micron-size devices (see Supplementary Figs S1 and S2 online) to emphasize the scalability of this technique.

**Figure 3:**
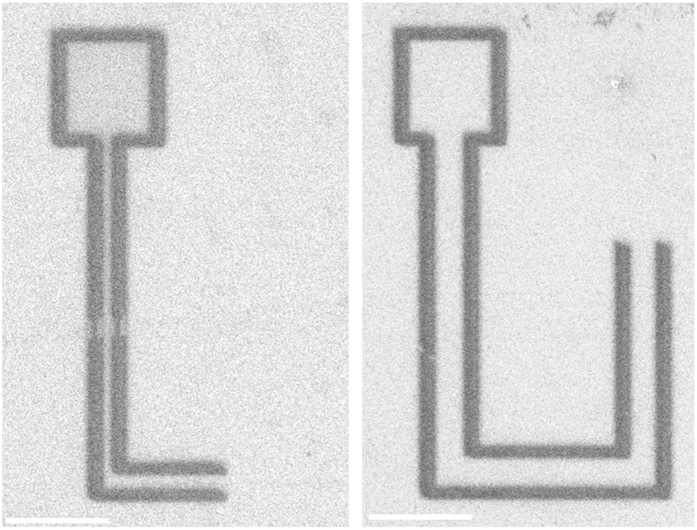
SHIM images of a graphene-based device that has been fabricated using direct-write Ne^+^ lithography. (Left panel) iSE image of conducting graphene pad with an L-shape conducting strip. The width of the conducting strip is ~100 nm. (Right panel) iSE image of conducting graphene pad with U-shape conducting strip. The width of the strip is ~250 nm. Scale bar is 1 μm.

Longer conducting graphene strips with different geometries were fabricated using the same method in order to investigate their effectiveness at longer length scales (micron length scale). Figure 3 displays supported graphene devices with longer strips that were fabricated using the Ne^+^ beam. Ne^+^ has a milling efficiency approximately 8 times greater per ion compared to He^+^ and can mill larger areas in a shorter time. The Ne^+^ beam was used in order to ensure that large structures were milled completely in the shortest time possible in order to prevent sample drift. Moreover, the positive charge of the Ne^+^ beam ensures a direct observation of the same positive charging effect in the graphene device. Although the geometry of the strip is nonlinear in Fig. 3 (left and middle panels), the pad remains conducting due to the absence of defects in the wire. The presence of defects in the wire leads to discontinuities in the conducting channel which would hinder electrons from getting to the graphene pad (see Supplementary Fig. S3 online). These images demonstrate that this approach may be used to fabricate large, complex and arbitrar*y* structures with immediate feedback concerning quality.

## Discussion

The minimum feature size achievable using SHIM is a product of several factors. There are the typical problems of instrumental drift and electronics stability in the raster scan. The SHIM described here is located in a cleanroom but not a low-noise microscopy suite, and undoubtedly experiences some mechanical noise. Also specific to this technique are the beam shape and ion backscattering which create a larger interaction volume between the beam and substrate than intended. The minimum width of the connecting strips reported here are likely determined by the density of defects along the edges of the strips, which are in turn determined by impingement by ions that escape outside of the illuminated region, either through an imperfect beam profile (tailing) or backscattering. Our experiments indicate that for a very short strip, the minimum width is about 10–12 nm. For longer strips, minimum width increases because the resistivity of strip is in part due to the sum of all defects along the length of the strip. This experiment has an additional limitation because as a focused ion beam technique, the quality of the device is dependent on operator skill in aligning and focusing the instrument.

Grain boundaries, which are a common occurrence in CVD-grown graphene can also affect the conductivity of graphene^12^,^13^. In our experiment, the graphene growth conditions^14^ were chosen to have graphene domains in excess of 100 μm, which is more than 2 orders of magnitude larger than the characteristic dimensions of the prepared devices. For some of the larger domains, the grain boundaries appear as wrinkles and folds and were avoided by taking a short dwell time (~0.5 μs) image of the area prior to milling. Smaller grains (less than 10 nm) could also be present within each fabricated device and will interrupt ballistic transport. These are not easily observed in the HIM due to imaging sensitivity limitations. Although these small-grain boundaries will impart some resistance, they are not insulating and are unlikely to affect the overall behavior of the fabricated devices because the behavior we are observing here does not depend entirely on ballistic transport but on overall conductivity. Lastly, it should also be noted that our graphene on SiO_2_ sample preparation method does not exclude the possibility of having contaminants from the residual PMMA resist that may be present after the transfer and annealing process (see Methods section). However, for non-CVD graphene, or any 2D material prepared without the need for transfer using a polymer, this approach may entirely exclude unnecessary exposure to solvents and resists.

In summary, we have demonstrated that positively charged ion beams from a SHIM may be used to fabricate devices in supported graphene on an insulator, and then detect conductivity in such devices *in situ*. The minimum width of the conducting strip attainable with scanning helium ion lithography is limited by the coupled vibrational modes arising from thermal fluctuations within the device. Electrical conductivity through a supported graphene strip is limited by the width of the strip. As a direct-fabrication technique, helium-ion lithography will be a preferred method of patterning graphene-based circuits and devices smaller or comparable in size to EBL, and will eliminate the need for the multistep processes involved in EBL.

## Methods

### CVD graphene on SiO_2_

Single layer graphene was synthesized using a method by Vlassiouk *et al.*^14^,^15^ Briefly, electropolished 125 μm thick copper foils were loaded into atmospheric pressure CVD reactor and annealed at 1065 °C under the flow of 2.5% H_2_ in Ar for 30 mins. Graphene growth was performed by addition of methane with a gradual increase in concentration from 10 to 20, to 40 ppm for 30 mins in each step. After growth, Microchem PMMA 495A4 solution was spin-coated at 2000 rpm on top of graphene on copper foil. Graphene from the back side of copper was etched away by oxygen plasma and copper was dissolved by 1 M FeCl_3_ in 3% HCl. Graphene/PMMA sandwich floating on water surface was washed by DI water and transferred onto the SiO_2_ substrate. PMMA was dissolved in acetone with subsequent annealing at 550 °C to remove the PMMA residue.

### Scanning Helium ion microscopy and lithography

Scanning helium ion microscopy and lithography on single layer graphene was performed using a Zeiss ORION Nanofab He/Ne ion microscope, operating at an accelerating voltage of 30 kV and a beam current of 4.2 pA. All the graphene devices were fabricated using the ion microscope’s built-in patterning software and imported bitmaps. Each milled area was exposed to the He^+^/Ne^+^ beam at a field of view and pixel spacing that yielded a fluence of ~1 × 10^19^ ions/cm^2^ for He^+^ beam and ~5 × 10^17^ ions/cm^2^ for the Ne^+^ beam. Subsequent high-resolution images were acquired at the same field of view using a 50 μs dwell time.

Scanning electron microscopy secondary electron images of the exact same graphene devices were obtained using a Zeiss MERLIN VP SEM equipped with an in-lens detector operating at 3 kV.

## Additional Information

**How to cite this article**: Iberi, V. *et al.* Maskless Lithography and *in situ* Visualization of Conductivity of Graphene using Helium Ion Microscopy. *Sci. Rep.* **5**, 11952; doi: 10.1038/srep11952 (2015).

## Supplementary Material

Supplementary Information
