# Supplementary material for: Maskless Lithography and in situ Visualization of Conductivity of Graphene using Helium Ion Microscopy
Source: Sci Rep. 2015 Jul 7;5:11952. doi: 10.1038/srep11952 (PMC4493665; doi:10.1038/srep11952)
Supplement: Supplementary Information [file srep11952-s1.pdf]

Maskless Lithography and *in situ* Visualization of Conductivity of Graphene using Helium Ion Microscopy

*Vighter Iberi[1], Ivan Vlassiouk[2], X.-G. Zhang[1,3], Brad Matola[1], Allyson Linn[1], David C. Joy[1,4] & Adam J. Rondinone[1]**

[1]Center for Nanophase Materials Science, Oak Ridge National Laboratory, Oak Ridge, TN 37831, USA, [2]Measurement Science & Systems Engineering, Oak Ridge National Laboratory, Oak Ridge, TN 37831, [3]Department of Physics and Quantum Theory Project, University of Florida, Gainesville, FL 32611, [4]Department of Materials Science & Engineering, University of Tennessee Knoxville, TN 37996

*Corresponding author: rondinoneaj@ornl.gov

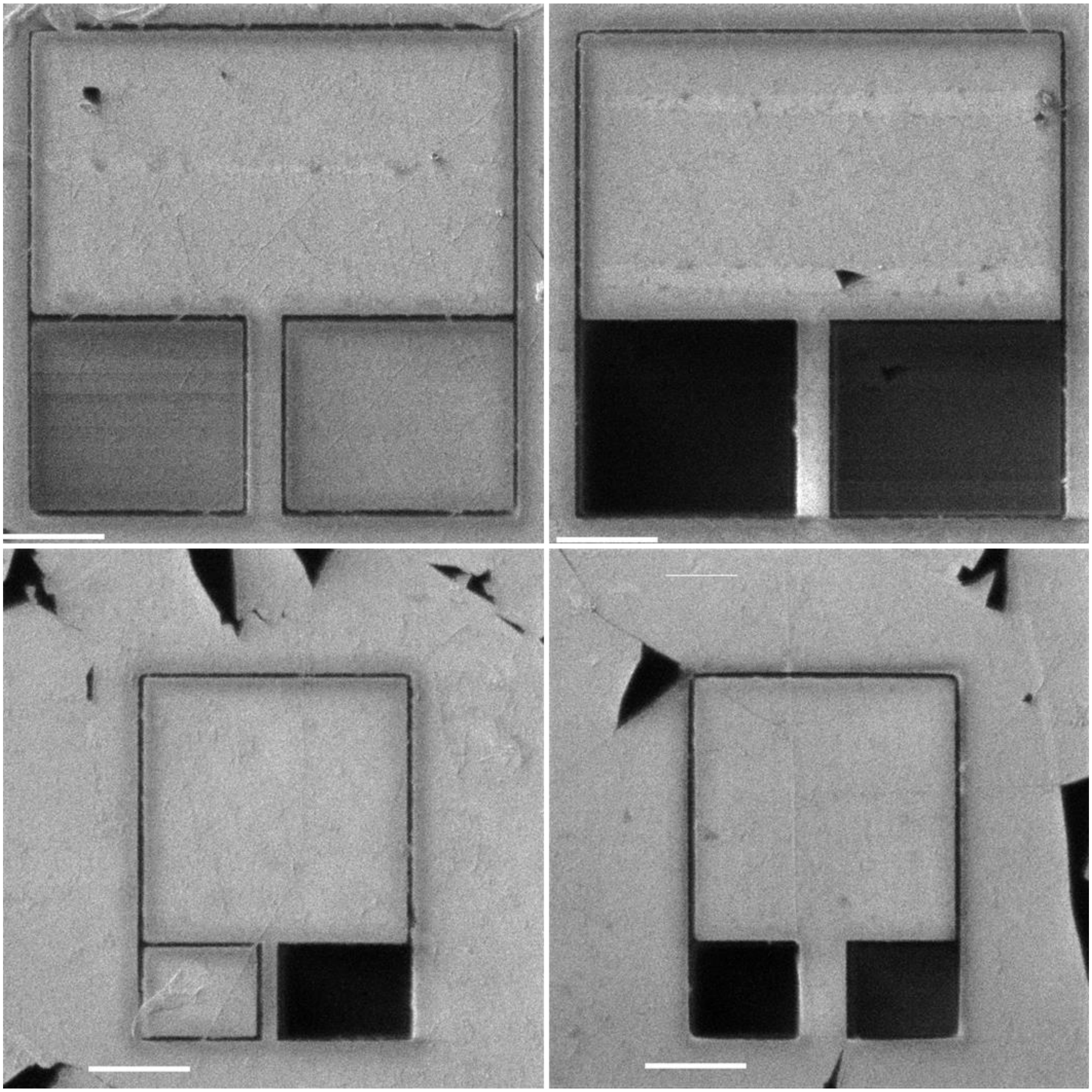

Supplementary Figure S1: SHIM images of micron-size graphene pads supported on SiO_2 that have been fabricated using direct-write He^+ lithography. Dark areas within each device indicate isolated graphene areas while areas outside the device that are dark indicate defects. Scale bar is 1 μm.

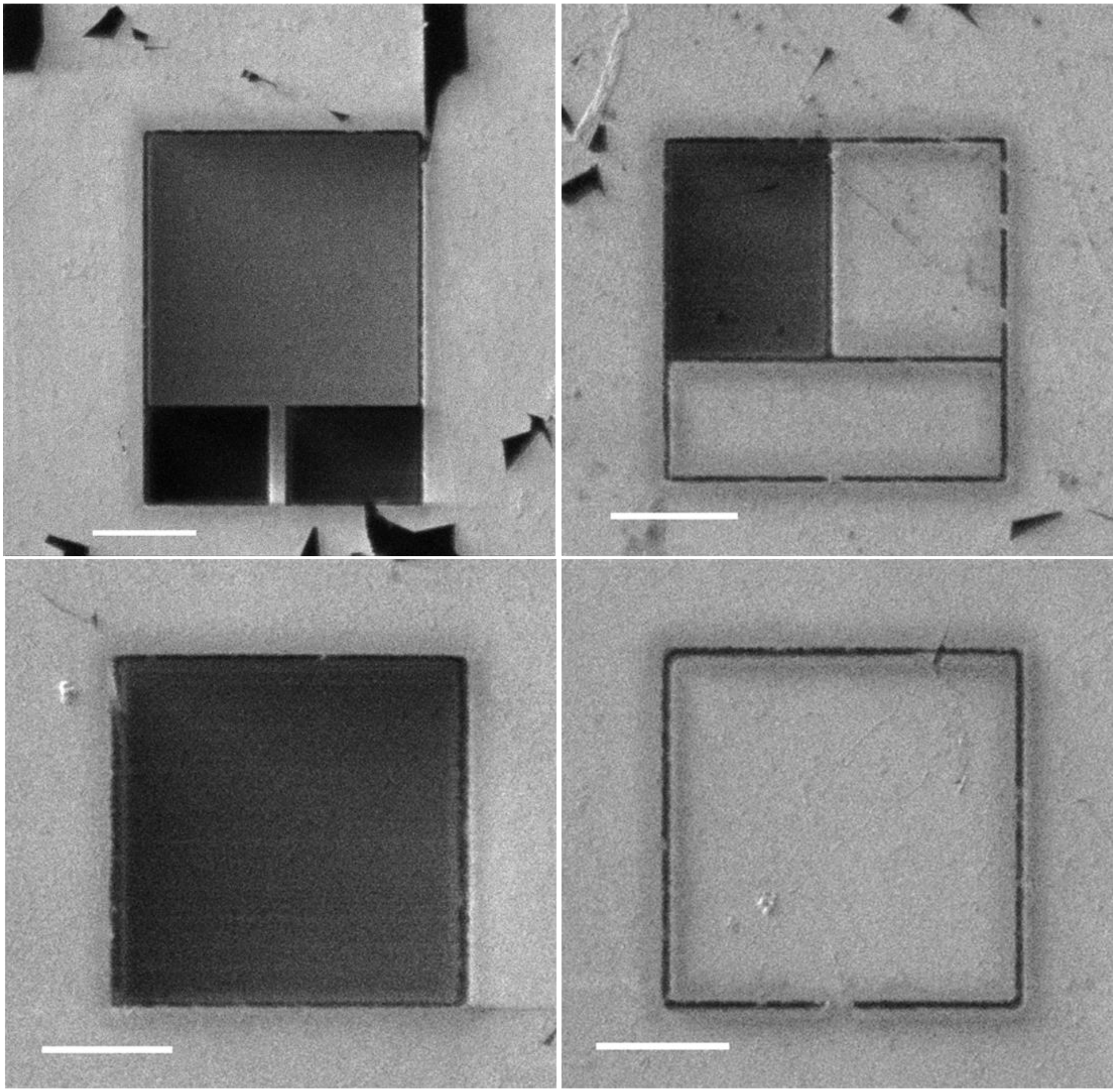

Supplementary Figure S2: SHIM images of micron-size graphene pads supported on SiO_2 that have been fabricated using direct-write He^+ lithography. Dark areas within each device indicate isolated graphene areas while areas outside the device that are dark indicate defects. Scale bar is 1 μm.

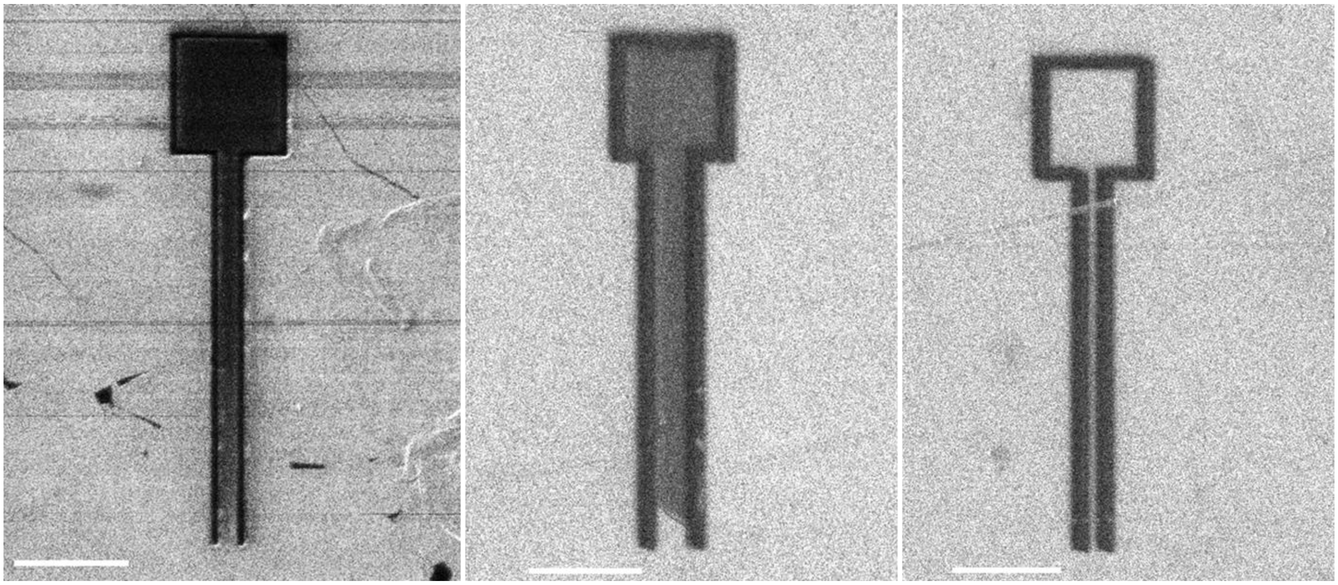

Supplementary Figure S3: iSE images of micron-size graphene pads supported on SiO_2 that have been fabricated using direct-write lithography. iSE image in the left panel was obtained using a Ne^+ beam while the images in the middle and right panels were obtained with a He^+ beam. Dark lines in Ne^+ beam iSE image are due to beam instability. Dark areas within each device indicate isolated graphene areas while areas outside the device that are dark indicate defects. Scale bar is 1 μm.
